# Prevalence of Malocclusions among Schoolchildren from Southwestern Romania

**DOI:** 10.3390/diagnostics14070705

**Published:** 2024-03-27

**Authors:** Stelian-Mihai-Sever Petrescu, Radu Mircea Pisc, Tamara Ioana, Felicia Ileana Mărășescu, Horia Octavian Manolea, Mihai Raul Popescu, Lucian Paul Dragomir, Lucian Constantin Dragomir, Ștefan Florea, Roxana Adina Bărăscu-Petrescu, Mihaela Ionescu, Anne-Marie Rauten

**Affiliations:** 1 Department of Orthodontics, Faculty of Dental Medicine, University of Medicine and Pharmacy of Craiova, 200349 Craiova, Romania; mihaipetrescu2702@gmail.com (S.-M.-S.P.); radu.pisc@gmail.com (R.M.P.); tamigmg@yahoo.com (T.I.); ciuca_felicia@yahoo.com (F.I.M.); rautenannemarie@yahoo.com (A.-M.R.); 2 Department of Dental Materials, Faculty of Dental Medicine, University of Medicine and Pharmacy of Craiova, 200349 Craiova, Romania; manoleahoria@gmail.com; 3 Department of Occlusology and Fixed Prosthetics, Faculty of Dental Medicine, University of Medicine and Pharmacy of Craiova, 200349 Craiova, Romania; popescumihairaul@yahoo.com (M.R.P.); dragomirlucianpaul@yahoo.com (L.P.D.); lucidragomir98@yahoo.com (L.C.D.); stefanflorea8@yahoo.com (Ș.F.); 4 Department of Prosthetics, Faculty of Dental Medicine, University of Medicine and Pharmacy of Craiova, 200349 Craiova, Romania; 5 Department of Medical Informatics and Biostatistics, Faculty of Dental Medicine, University of Medicine and Pharmacy of Craiova, 200349 Craiova, Romania

**Keywords:** Angle’s classification, dysfunction, malocclusion, mixed dentition, parafunction, prevalence, statistical analysis

## Abstract

Malocclusions have a continuously increasing prevalence from one generation to another as a result of climate change, soil, atmosphere, and water pollution. All of these aspects have unfavorable consequences for the nutritional scheme. Thus, nutrition, together with other etiopathogenic factors, contributes to complex alterations in the somatic development of the entire organism and, implicitly, of the cephalic extremity. The study group included 4147 children from randomly selected schools from Vâlcea County, Romania. The aim of this study is to determine the prevalence of malocclusions in schoolchildren in Vâlcea County, Romania, according to the three main classes of malocclusions (according to Angle’s classification), age groups (from 6 to 10 years old and from 11 to 14 years old), gender (male and female), and place of origin (rural and urban). For Angle class I malocclusions, we recorded the highest prevalence (48.78% of the total number of schoolchildren with malocclusions), followed by Angle class II malocclusions (45.85% of the total number of schoolchildren with malocclusions), and for Angle class III malocclusions we found the lowest prevalence (5.37% of the total number of schoolchildren with malocclusions). According to gender, we found the highest prevalence in the female gender (29.90% of the total number of female subjects), while in the male gender, we recorded a prevalence of 27.70% of the total number of male subjects. Regarding the place of origin, there is a higher prevalence of malocclusions in urban areas (29.16%). The study subgroup included 140 children randomly selected from the total number of subjects in the study group. They were included in a more advanced study. The aim is to find potential associations between the presence of malocclusions and various oral variables. Categorical variables were expressed as numerical values and percentages, and their association was evaluated with either the Chi-square test of association or homogeneity, or the Fisher Exact test. The acquired data were incorporated into a binomial logistic regression model to assess the likelihood of developing malocclusions in relation to the following variables: defective phonation, bruxism, frequency of teeth brushing, onychophagia, oral respiration, infantile deglutition, placing objects between the maxillaries, thumb sucking, and salivary aspects. It is also aimed at comparing the results obtained with similar ones from the specialized literature.

## 1. Introduction

Malocclusions represent imbalances that appear in the formation and growth processes of the stomatognathic system [1]. Malocclusions affect the functions of the dentomaxillary apparatus (respiratory function, mastication, deglutition, phonation, and physiognomy), the patient’s psycho-social integration, and, implicitly, the quality of his life [2,3,4]. Thus, understanding malocclusion from the patient’s perspective is crucial in orthodontic treatment planning. Since perceptions of malocclusion can vary among individuals and cultural backgrounds, incorporating subjective measures alongside professional assessments helps provide a comprehensive understanding of the patient’s needs and priorities [5].

Globally, the most common type is the Angle class I malocclusions, which vary greatly between nations (31% in Belgium and 96.6% in Nigeria). Africans (89.44%) have a higher prevalence than Caucasians (71.61%) and Asians (74.87%), according to the distribution pattern. Furthermore, Asians have a rise in Angle class I malocclusions during puberty, indicating that developmental factors may be involved in this population’s susceptibility to malocclusions at particular life phases. The global prevalence of Angle class II malocclusions is 19.56%, although there is a significant variance between nations, ranging from 1.6% in Nigeria to 63% in Belgium. With a recorded prevalence of 22.9%, Caucasians are the most affected, followed by Asians (14.14%) and Africans (6.76%). In the two sets of teeth, the worldwide distribution pattern of Angle class II malocclusions by race is rather comparable. With the exception of Africans, there is a propensity for correction of Angle class II malocclusions throughout pubertal growth, particularly in the transition from mixed to permanent dentition. The genetic effects can be attributed to both the occurrence and treatment of Angle class II malocclusions resulting during pubertal growth. According to data, the prevalence of Angle class III malocclusions is 5.93% overall, although the numbers vary by country, from 0.7% in Israel to 19.9% in China. The reported prevalence for Asians is 9.63%, Africans 3.8%, and Caucasians 5.92%. In both mixed and permanent dentitions, the worldwide distribution model of Angle class III malocclusions appears to be stable. Notably, in both Caucasians and Africans, there is a tendency for the development of Angle class III malocclusions to rise during the shift from mixed to permanent dentition. An important focus in the genesis of Angle class III malocclusions is the hereditary component. In Asians, these malocclusions are mainly caused by developmental deficits in the middle third of the face rather than mandibular prognathism [1].

In developed countries, preventive dentistry occupies an important place in national public health programs. In Romania, the National Health Insurance House (NHIH) is not involved in promoting prevention, which has led to an alarming increase in the prevalence of malocclusions. The implementation of prophylaxis in orthodontics can eliminate the etiopathogenic factors, preventing the occurrence of dentomaxillary apparatus disorders or allowing their treatment in the initial stages. The prevention of malocclusions leads to the reduction of the period reserved for active orthodontic therapy or even to its complete elimination [6,7,8].

Currently, the World Health Organization (WHO) estimates that malocclusions are the third most common oral health problem, after dental caries and periodontal diseases [9,10,11].

In this regard, there has been a continuous increase in critical reviews of the accuracy of the results of published epidemiological research. Their purpose is to generate accurate evidence of the health problem under investigation using strict criteria for assessing quality. However, few such studies have been conducted in orthodontics.

Regional epidemiological assessment of malocclusions is extremely important, as it provides useful data to establish the type and distribution of occlusal characteristics. This information will help prioritize the importance of orthodontic treatment and determine the resources needed [12,13]. In addition, establishing the prevalence of these anomalies in several population groups in different locations may reflect the existence of genetic and environmental determinants [14,15].

Therefore, the availability of this data will be important for educational purposes, advancing research, informing public health efforts, promoting early intervention, addressing health disparities, and improving the quality of orthodontic care for patients.

## 2. Materials and Methods

The present study was approved by the Ethics Committee of the University of Medicine and Pharmacy of Craiova, Romania (approval reference no. 72/07.09.2020), in accordance with the ethical guidelines for research with human participants at the University of Medicine and Pharmacy of Craiova, Romania. Informed consent was obtained from all the legal guardians of the subjects involved in the study.

We conducted a cross-sectional epidemiological study to determine the prevalence of malocclusions in the population of Vâlcea County, located in southwestern Romania. The research was carried out through a partnership with 4 randomly selected schools from Vâlcea County, Romania, and related School Inspectorates.

To fulfill the proposed objectives, we took into account the following criteria for inclusion in the study: children’s age must be between 6 and 14 years old; the children must be students of the selected educational units; informed consent must be obtained from the legal guardians of the children; the children must give their verbal consent for examination; the participation must be voluntary, and the children must be cooperative.

We excluded from the study: children below 6 and above 14 years old; children for whom a legal guardian did not provide informed consent; children with a psychiatric condition; and those who are not willing to be examined (uncooperative children).

Therefore, following the application of these criteria, we obtained the study group, consisting of 4147 schoolchildren.

The children were examined in the school medical offices, using natural light. The clinical examination consisted of visualizing the oral cavity using disposable dental consultation kits. During the clinical examination, the child was sitting on a chair in front of the doctor, who was also sitting on a chair. The data obtained after the examination were recorded in a file, which included: age, gender, place of origin, dysfunctions, parafunctions, and orthodontic diagnosis.

In our study, only children with mixed dentition were examined. This period is an important developmental stage for the undisturbed occlusal relationship. The eruption and evolution of the first permanent molars play an important role in maintaining the interarch space and the sagittal-occlusal relationship. A large number of malocclusions occur during the period of mixed dentition [16,17]. The evidence from the specialized literature has indicated that early intervention starting with the mixed dentition would benefit children with malocclusions, such as Angle class III malocclusions, crossbites, and crowding [18,19,20,21].

The complexity of malocclusions and their multifactorial etiopathogenesis have generated numerous classifications. However, none of the classifications used in orthodontics are complex enough. In our study, we opted for Angle’s classification, the first that was stated in 1899 and the most used globally even nowadays. It is considered that the first permanent upper molars have fixed positions, and thus the mesiodistal relations they establish with the first permanent lower molars are followed. Angle’s class I is characterized by a neutral relationship between the first permanent molars, which is concretized by the alignment between the most mesial intercuspal groove from the buccal surface of the first permanent lower molar and the tip of the mesiobuccal cusp of the first permanent upper molar. In Angle’s class II are included malocclusions with a distalized relation of first permanent molars, being described by a distal placement of the most mesial intercuspal groove from the buccal surface of the first permanent lower molar compared to the tip of the mesio-buccal cusp of the first permanent upper molar. Angle’s class III is characterized by a mesialized relation of first permanent molars, being described by a mesial placement of the most mesial intercuspal groove from the buccal surface of the first permanent lower molar compared to the tip of the mesio-buccal cusp of the first permanent upper molar [22]. Of the total number of 4147 schoolchildren, 140 children were randomly selected among the cooperative patients who could be subjected to a more advanced study regarding the presence of malocclusions and potential associations with various oral variables. Half of this group represented children with malocclusions (the M group), while the other half represented the control group.

For this subgroup study, the following variables were acquired by two examiners: oral respiration, infantile deglutition, level of somatic development, defective phonation, slow mastication, bruxism, salivary aspect, smoking status, consumption of acid drinks, thumb sucking, onychophagia, placing objects between the maxillaries, consumption of seeds, and frequency of teeth washing.

The data obtained from the clinical examination were processed and recorded electronically with the help of the software package Microsoft Excel 365 (Microsoft Corp., Redmond, WA, USA). Thus, the results of our study could be presented through representative graphs. Categorical variables were expressed as numerical values and percentages, and their association was evaluated with either the Chi-square test of association or homogeneity or the Fisher Exact test. The measure of inter-rater agreement for categorical scales was determined using the Cohen Kappa value. All statistical tests were employed using Statistical Package for Social Sciences (SPSS), version 26 (IBM Corp., Armonk, NY, USA). The acquired data were incorporated into a binomial logistic regression model to assess the likelihood of developing malocclusions in relation to the following variables: gender, residence, defective phonation, bruxism, frequency of teeth brushing, onychophagia, oral respiration, infantile deglutition, placing objects between the maxillaries, thumb sucking, and salivary aspects. The α threshold was set to 5%, and *p* < 0.05 was considered statistically significant.

## 3. Results

### 3.1. General Characteristics of the Study Group

**Figure 1:**
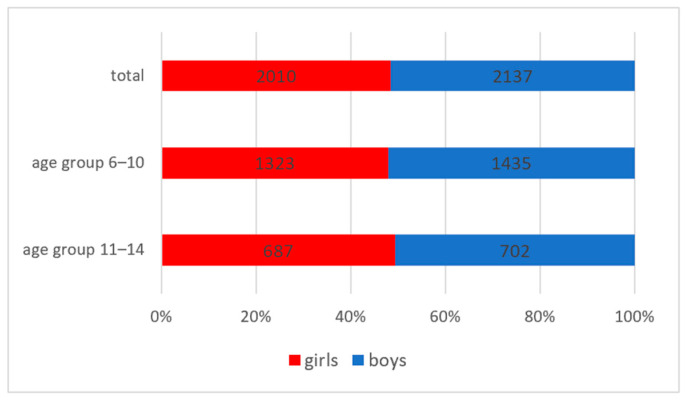
Distribution of the studied group according to age group and gender.

The study included 4147 schoolchildren, aged between 6 and 14 years old. In the 6–10 age group, 1323 were girls and 1435 were boys. In the 11–14 age group, 687 were girls and 702 were boys (see Figure 1).

**Figure 2:**
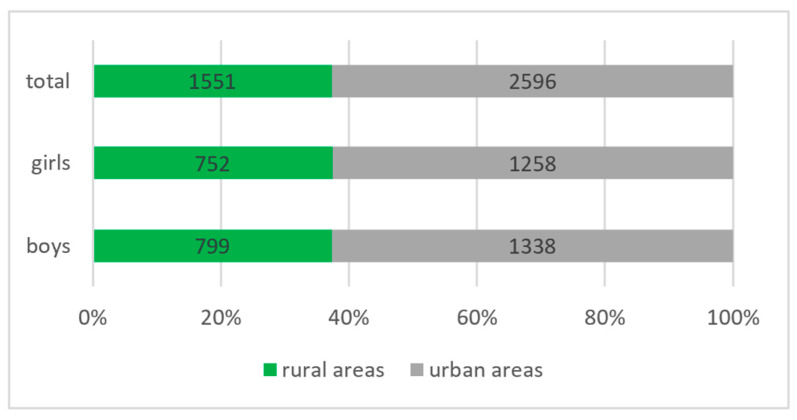
Distribution of the studied group according to the place of origin and gender.

From the studied group, 1551 schoolchildren came from rural areas, of which 752 were girls and 799 were boys; 2596 schoolchildren came from urban areas, of which 1258 were girls and 1338 were boys (see Figure 2).

### 3.2. Distribution of Malocclusions

**Figure 3:**
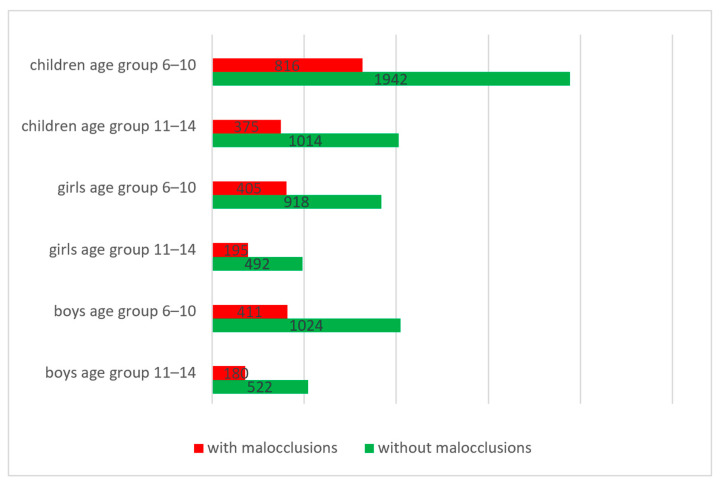
Distribution of malocclusions according to age group and gender.

From the total of 4147 subjects included in the study, in the age group 6–10, we identified 816 schoolchildren with malocclusions, of which 405 were girls and 411 were boys. In the age group 11–14, we found 375 schoolchildren with malocclusions, of which 195 were girls and 180 were boys. The remaining 2956 subjects did not present malocclusions (see Figure 3).

We recorded the highest number of schoolchildren with malocclusions in the female gender, Angle class I (315). We found the lowest number of schoolchildren with malocclusions in the female gender, Angle class III (29) (see Table 1).

In the rural areas of Vâlcea County, we recorded the highest number of schoolchildren with malocclusions in female gender, Angle class I (120). We found the lowest number of schoolchildren with malocclusions in the male gender, Angle class III (11) (see Table 2).

In the urban areas of Vâlcea County, we recorded the highest number of schoolchildren with malocclusions in the female gender, Angle class I, and in the male gender, Angle class II (195 each). We found the lowest number of schoolchildren with malocclusions in the female gender, Angle class III (17) (see Table 3).

**Figure 4:**
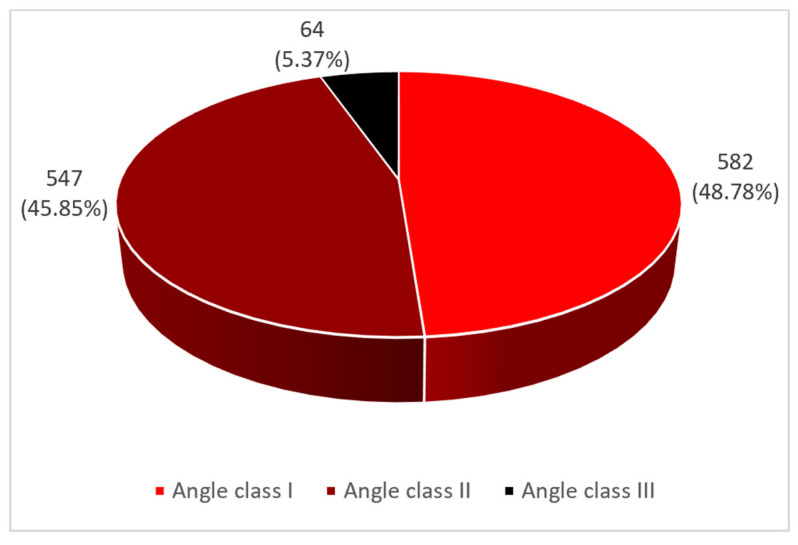
Prevalence of malocclusions according to Angle’s classification.

For Angle class I malocclusions we recorded the highest prevalence (48.78% of the total number of schoolchildren with malocclusions), followed by Angle class II malocclusions (45.85% of the total number of schoolchildren with malocclusions), and for Angle class III malocclusions we found the lowest prevalence (5.37% of the total number of schoolchildren with malocclusions) (see Figure 4).

**Figure 5:**
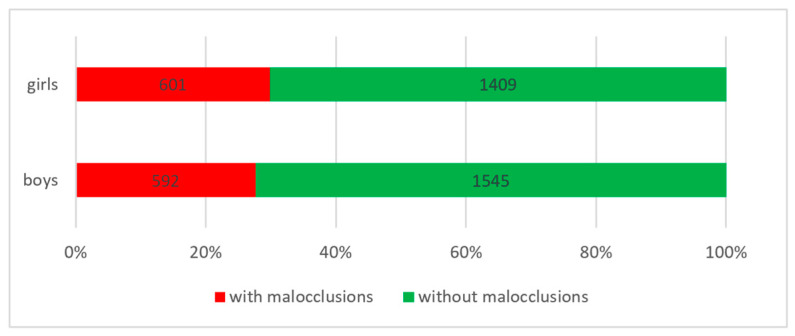
Prevalence of malocclusions according to gender.

According to gender, we found the highest prevalence in the female gender (29.90% of the total number of female subjects), while in the male gender, we recorded a prevalence of 27.70% of the total number of male subjects (see Figure 5).

**Figure 6:**
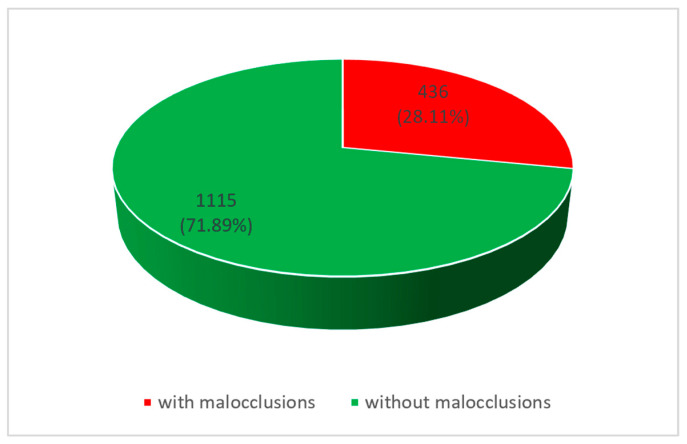
Prevalence of malocclusions in rural areas.

In the rural areas, we found a prevalence of malocclusions of 28.11% (see Figure 6).

**Figure 7:**
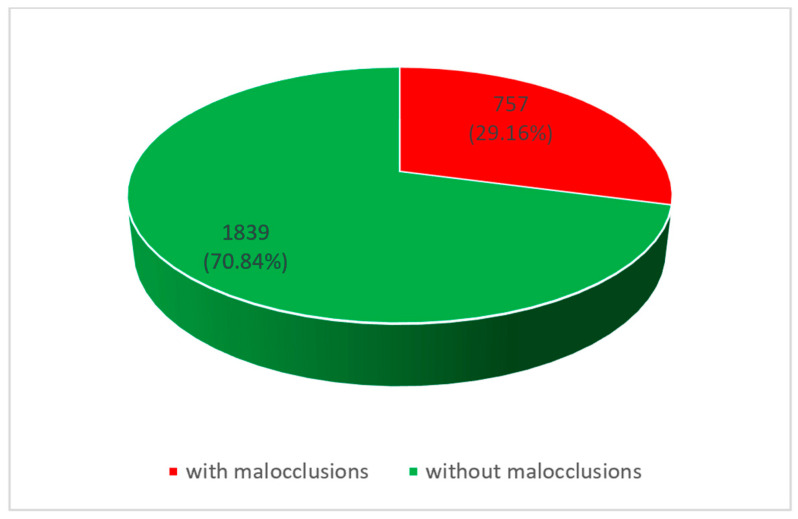
Prevalence of malocclusions in urban areas.

In the urban areas, we found a prevalence of malocclusions of 29.16% (see Figure 7).

**Figure 8:**
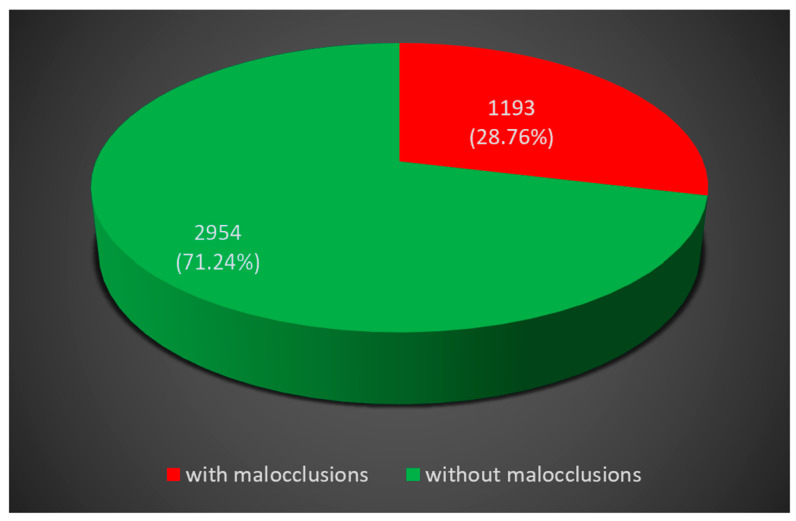
Prevalence of malocclusions in Vâlcea County, Romania.

In Vâlcea County, Romania, we recorded a prevalence of malocclusions of 28.76% (see Figure 8).

### 3.3. Subgroup Study

Cohen’s Kappa index was calculated to determine the index of agreement between the two examiners on the analysis of the somatic development level and the salivary aspects for a sample of 30 children.

Regarding the level of somatic development, the two examiners agreed on 19 children with normal development, 6 children with a lower level of somatic development, and 2 children with a higher level of somatic development. However, examiner 1 rated 2 children as having a normal level, while Examiner 2 rated them as having a lower level, respectively a higher level of somatic development. Also, examiner 1 rated 1 child as having a lower level, while examiner 2 rated the child as having a normal level of development. Overall, there was a good level of agreement between the analysis of the two medical examiners, κ = 0.788, *p* < 0.0005.

Regarding the salivary aspects, the two examiners agreed on 19 children with a normal level of secretion, 7 children with hyposecretion, and 2 children with hypersecretion of saliva. Thus, examiner 1 rated 1 child as having a normal level of saliva secretion, while examiner 2 rated the child as having a hyposecretion. Also, examiner 1 rated 1 child as having hypersecretion, while examiner 2 rated the child as having a normal level. Overall, there was a very good level of agreement between the two examiners’ analyses, κ = 0.863, *p* < 0.0005.

**Figure 9:**
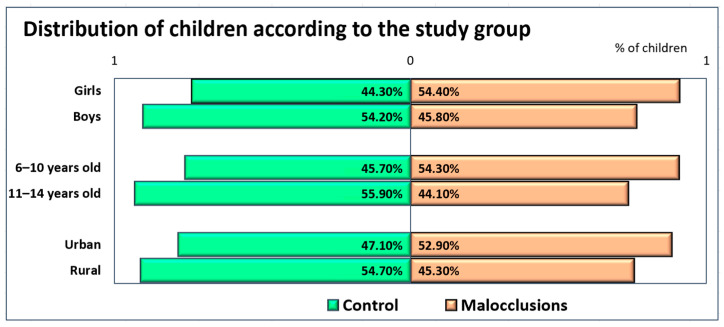
Distribution of children according to study group, gender, age group, and place of origin.

The study lot comprised 68 girls (representing 48.57% of the entire lot) and 72 boys (51.43%), divided by age as follows: 81 children (57.86%) with ages between 6 and 10 years old, and 59 children (42.14%) with ages between 11 and 14 years old. More than half of the children had urban residence, 40 girls (58.8% of all girls) and 47 boys (65.3% of all boys), while 28 girls 41.2%) and 25 boys (34.7%) had rural residence. Figure 9 emphasizes the distribution of the study group according to gender, age group, and place of origin.

A chi-square test for association was conducted between the presence of malocclusions and demographic data (gender, age group, and place of origin). All expected cell frequencies were greater than five. There were no statistically significant associations between the presence of malocclusions and the demographic data, χ^2^ (1) = 1.029, *p* = 0.310 (for gender), χ^2^ (1) = 1.435, *p* = 0.231 (for age groups) and χ^2^ (1) = 0.759, *p* = 0.384 (for the place of origin).

Approximately half of the children included in the study lot brushed their teeth once a day, divided as follows: 55.6% of children from the age group 6–10 years old, and 47.5% of children ages above 11 years old. Almost a third of smaller children never brush their teeth, compared to only 13.6% of older ones. Children above 11 years old are more aware of the need to brush several times a day, as 28.8% brush their teeth twice a day, and 10.2% even three times a day. Thus, the differences between the age groups relative to the frequency of brushing teeth are statistically significant, χ^2^ (3) = 14.655, *p* = 0.002.

#### 3.3.1. Oral Parameters’ Analysis

Oral respiration has been identified in 10 children with rural residence (representing 18.9% of all children from the countryside), compared to only 6 children with urban residence (representing 6.9% of all children from urban areas), and these differences are statistically significant (χ^2^ (1) = 4.663, *p* = 0.031). Also, this type of respiration is associated with a low level of somatic development (χ^2^ (2) = 59.756, *p* < 0.0005), with 87.5% of children within this level being characterized by oral respiration, compared to 8.9 children with normal respiration. The rest of the 12.5% of children have a normal level of development, and no child has a high level of development. Similar results have been identified for the salivary aspect, as 81.3% of children with oral respiration have a low level of saliva secretion (χ^2^ (2) = 62.246, *p* < 0.0005). More than half of all children with oral respiration have a defective phonation (62.5%), thus there is a positive association that is statistically significant between these parameters (χ^2^ (1) = 27.928, *p* < 0.0005). Other two parameters are associated with oral respiration: thumb sucking (specific for 68.8% of children with oral respiration) and placing objects between maxillaries (specific for 62.5% of children with oral respiration), with χ^2^ (1) = 58.637, *p* < 0.0005, respectively χ^2^ (2) = 19.205, *p* < 0.0005.

All children with infantile deglutition have a defective phonation, therefore there is a significant association between these variables, χ^2^ (1) = 31.888, *p* < 0.0005. Half of these children have a low level of secretion, while the other half is characterized by a normal level, and none with hyper-secretion, the differences being statistically significant, χ^2^ (2) = 6.174, *p* = 0.046. Infantile deglutition is also associated with placing objects between maxillaries (specific for 83.3% of children with infantile deglutition), with χ^2^ (1) = 14.967, *p* < 0.0005.

More than half of all children identified with thumb sucking (62.5%) have a low level of somatic development and a low level of saliva secretion, thus leading to a significant association between these variables, χ^2^ (2) = 24.775, *p* < 0.0005, respectively χ^2^ (2) = 32.212, *p* < 0.0005. Defective phonation is also associated with thumb sucking, with 56.3% of children with thumb sucking having phonation issues, χ^2^ (1) = 20.864, *p* < 0.0005, and with placing objects between the maxillaries, with 81.3% of children with thumb sucking having this behavior, χ^2^ (1) = 40.307, *p* < 0.0005.

The majority of children identified with slow mastication (69.2%) have a low level of somatic development and a normal level of saliva (61.5%), and no high levels for any of these two parameters, thus there are statistically significant differences between these variables, χ^2^ (2) = 25.908, *p* < 0.0005, respectively χ^2^ (2) = 6.709, *p* = 0.035.

Children with bruxism have mostly a normal level of saliva secretion (82.4%), but 17.6% have hyper-secretion, thus the differences between these parameters are statistically significant, χ^2^ (1) = 6.726, *p* = 0.035.

Two-thirds of children (66.7%) with a hypo-secretion of saliva are also characterized by a low level of somatic development, and 33.3% of children with saliva hyper-secretion have a high level of somatic development, thus there are statistically significant differences between salivary aspects and somatic development, χ^2^ (4) = 53.292, *p* < 0.0005. Also, a quarter of children with saliva hypo-secretion are aged between 11 and 14 years old, compared with only 7.4% of children aged between 6 and 10 years old. Normal secretion was identified for 85.2% of children aged below 10, and only 69.5% of children aged above 10. The differences between age groups regarding the salivary aspects are statistically significant, χ^2^ (2) = 8.743, *p* = 0.013.

Onychophagia was identified in 20.7% of all children, 34.5% of them had saliva hypo-secretion, 6.9% had hyper-secretion, and the rest had normal levels. Most children without onychophagia had normal levels of saliva, therefore the differences between these variables are statistically significant, χ^2^ (2) = 11.121, *p* = 0.004.

The habit of placing objects between the maxillaries was encountered in 29 children (representing 20.7% of the entire study lot). Around half of them (55.2%) had a normal level of saliva secretion, compared to 84.7% of children without this habit, and 41.4% had hypo-secretion, compared to 8.1% from the opposite group, thus statistically significant differences were identified between these groups, χ^2^ (2) = 20.022, *p* < 0.0005. Similar percentages were identified regarding the level of somatic development, χ^2^ (2) = 10.100, *p* = 0.006.

#### 3.3.2. M and Control Groups Comparison

Oral respiration (observed in 16 children, 11.43% of the entire study lot), infantile deglutition (6 children, 4.3%), thumb sucking (16 children, 11.43%), and placing objects between the maxillaries (29 children, 20.7%) were diagnosed only in children from the M group. For all these parameters, the differences between the two study groups are statistically significant (*p* < 0.0005) (Table 4).

The analysis of children’s somatic development identified a low level for almost 18% of the study lot (25 children), with two-thirds of them being included in the M group and one-third in the control group. A normal level was identified for 107 children, with a similar distribution between the two study groups, while a high level was identified for 8 children, equally distributed between the M and control groups. The differences between the two groups were not statistically significant, *p* = 0.136 (Table 4). Similar results were also obtained for slow mastication, as only 13 children presented this sign, with 61.5% of them in the M group and 38.5% in the control group, *p* = 0.382 (Table 4).

The presence of bruxism is another sign that is not directly related to the presence of malocclusions; 17 children have this disease, almost two-thirds being included in the M group and the rest in the control group, but no statistically significant differences were identified between the two groups (Table 4).

Smoking status and dietary factors (consumption of acid drinks and consumption of seeds) are habits that have been identified in both groups, in rather small percentages, with similar distributions and no statistically significant differences between groups (Table 4).

For the 70 children within the M group, the distribution by class was the following: 34 children (48.6%) had class I, 30 children (42.9%), and only 6 children (8.6%) had an Angle class III malocclusion. There were no statistically significant differences between these three classes relative to the demographic data (gender, age groups, and place of origin), *p* > 0.05. The distribution of oral parameters by the malocclusion class is presented in Table 5.

All children with oral respiration had Angle class II malocclusions, as well as all children with thumb sucking habit, while all children with infantile deglutition had only Angle class I malocclusions, therefore the differences between classes regarding these variables were statistically significant, *p* < 0.05 (Table 5). The majority of children with a low level of somatic development have Angle class II malocclusions, while those with a high level have Angle class III malocclusions; the normal level is mostly defined by Angle class I malocclusions, therefore the differences between classes are statistically significant, *p* < 0.0005 (Table 5). Similar associations were identified for the level of saliva secretion, *p* = 0.006 (Table 5).

Almost all children with bruxism have Angle class I malocclusions, while those without bruxism have Angle class II malocclusions, differences being statistically significant, *p* = 0.009. A similar distribution was identified for the consumption of acid drinks, as children who consume this kind of drinks mostly have Angle class I malocclusions, while those who do not have Angle class II malocclusions, *p* < 0.0005 (Table 5).

For the following parameters: defective phonation, slow mastication, smoking status, onychophagia, the habit of placing objects between the maxillaries, consumption of seeds, and frequency of teeth brushing, there were no significant differences between classes, *p* > 0.05 (Table 5).

#### 3.3.3. Logistic Regression

A binomial logistic regression was performed to ascertain the effects of gender, place of origin, defective phonation, bruxism, frequency of teeth brushing, onychophagia, oral respiration, infantile deglutition, placing objects between the maxillaries, thumb sucking and salivary aspects on the likelihood that children present malocclusions. These parameters were chosen based on their statistical significance associated with the presence of malocclusions. There was one standardized residual with a value of 3.194 standard deviations, which was kept in the analysis. The logistic regression model was statistically significant, χ^2^ (14) = 91.584, *p* < 0.0005. The model explained 64.0% (Nagelkerke R^2^) of the variance in heart disease and correctly classified 84.3% of cases. Sensitivity was 77.1%, specificity was 91.4%, positive predictive value was 90.0% and negative predictive value was 80.0%. Only gender, place of origin, and no teeth brushing were statistically significant. Thus, girls have higher odds of developing malocclusions compared to boys, and children living in urban areas compared to rural areas, as well as children who never brush their teeth.

## 4. Discussion

In Romania, there are few up-to-date data on the prevalence of malocclusions on a large number of subjects and geographical regions.

In 2021, Petrescu et al. conducted a cross-sectional study on 1007 children from Gorj County, Romania, also located in the southwest of the country. The researchers recorded the highest prevalence for Angle class I malocclusions (63.20%) and the lowest prevalence was recorded for Angle class III malocclusions (4.94%) [7].

In 2022, Petrescu et al. performed another epidemiological research on 1255 children from Olt County, also located in southwestern Romania. The authors reported the highest prevalence for Angle class I (51.18%) and the lowest prevalence was found for Angle class III malocclusions (7.08%) [8].

Research held in the mining areas of the Apuseni Mountains, Romania found the same distribution of malocclusions: 56.4% for Angle class I, 37.9% for Angle class II, and 5.70% for Angle class III [6].

A statistical study on the prevalence of malocclusions was performed in the Dental Ambulatory for Children from Iaşi, Romania, between 2000 and 2010, on a number of 375 children aged between 4 and 24 years old. The researchers identified the highest prevalence for Angle class I malocclusions (63.2%) and the lowest for Angle class III malocclusions (5.8%) [23].

Epidemiological studies on the prevalence of malocclusions have also been carried out in other European countries.

Thus, a prevalence of 47.30% for Angle class I malocclusions, 45.10% for Angle class II malocclusions, and 5.40% for Angle class III malocclusions was found on Hvar Island, Croatia [24].

Research carried out in Hungary identified the highest prevalence for Angle class I malocclusions (52.80%), followed by Angle class II malocclusions (39.10%) and Angle class III malocclusions (8.10%) [25].

Another study of this type was conducted in southern Italy and found a prevalence of 59.50% for Angle class I malocclusions, 36.30% for Angle class II malocclusions, and 4.20% for Angle class III malocclusions [26].

In Sweden, an epidemiological analysis found the highest prevalence for Angle class I malocclusions (64%), followed by Angle class II malocclusions (25.80%) and Angle class III malocclusions (10.20%) [27].

In another study, the researchers focused on two issues that are common in the pediatric population: food selectivity and autism spectrum disorder (ASD). 111 children between 2 and 10 years old composed the study group. Of them, 60 have been diagnosed with autism, while the remaining 51 have neurotypical development, which is characterized by similar traits but without the condition. The results of this research provide evidence of eating and food selectivity difficulties. Furthermore, this study shows that sensory issues pertaining to food texture, color, smell, and warmth as well as severe sensory modulation can contribute to food selectivity. Actually, the findings highlight the relationship between food choice and the smell and taste senses. Additionally, this study demonstrates a relationship between eating abilities and motor skills, specifically with regard to food selectivity, which is strongly linked to irregular and disruptive eating patterns [28].

The strong points of this research were the large number of subjects that formed the study group and, at the same time, the homogeneity of the study group. In a regional epidemiological assessment of malocclusions, all the data are collected from the same population, while in a systematic review, different populations (with heterogeneous characteristics) are used for collecting data.

Even though regional epidemiological studies play an essential role in understanding the burden of malocclusions within specific geographic areas, they have some limitations. Regional epidemiological studies struggle to obtain a sample that accurately represents the entire population within the region. Factors such as the sampling method used, recruitment strategies, and participant demographics influence the representativeness of the sample. If certain demographic groups are underrepresented in the sample, the prevalence estimates may not be generalizable to the entire population. Also, findings from regional epidemiological studies are not easily extrapolated to other regions due to differences in population demographics, healthcare systems, and cultural practices. Caution should be exercised when generalizing findings from regional studies to other geographic areas.

In the future, evaluating and developing preventive strategies will help to reduce the incidence and severity of malocclusions, particularly in children. Community-based interventions, such as oral health education programs, early orthodontic screening initiatives, and school-based dental sealant programs, could help prevent or mitigate malocclusion-related problems.

## 5. Conclusions

An analysis of the articles in the literature showed that, of the different classes of malocclusions described by Angle, class I is the most frequent, followed closely by class II and finally by class III. A further useful investigation could concern the subdivisions of the second class, which was little taken into account.

The prevalence of malocclusions according to Angle’s classification varied widely, considering different countries and, sometimes, even within the same country but in distinct geographical regions. This suggests an important role for environmental factors. Knowing the prevalence of malocclusions within a region is important and useful for establishing public health policies in orthodontics.

In our study, we found a prevalence of malocclusions of almost 30%, which did not vary significantly with age, gender, or residence.

However, compared to other studies cited in the literature, in our study, we observed an increased prevalence for Angle class II malocclusions and a lower one for Angle class I, both compared to other studies from Romania and also from other European countries.

In Romania, the ever-increasing prevalence of malocclusions from one generation to another suggests a lack of prevention and reduced addressability of patients for orthodontic treatment.

At the same time, the data gathered from the studies on the prevalence of malocclusions must represent an alarm signal in order to establish public health policies in orthodontics.

## Figures and Tables

**Table 1:** Distribution of malocclusions according to Angle’s classification and gender.

	Cases		%	
	Female	Male	Female	Male
Angle class I	315	267	52.41%	45.10%
Angle class II	257	290	42.76%	48.98%
Angle class III	29	35	4.83%	5.92%
Total	601	592	100%	100%

**Table 2:** Distribution of malocclusions in rural areas according to Angle’s classification and gender.

	Cases		%	
	Female	Male	Female	Male
Angle class I	120	102	52.63%	49.03%
Angle class II	96	95	42.10%	45.67%
Angle class III	12	11	5.27%	5.30%
Total	228	208	100%	100%

**Table 3:** Distribution of malocclusions in urban areas according to Angle’s classification and gender.

	Cases		%	
	Female	Male	Female	Male
Angle class I	195	165	52.27%	42.96%
Angle class II	161	195	43.16%	50.78%
Angle class III	17	24	4.57%	6.26%
Total	373	384	100%	100%

**Table 4:** Distribution of children by oral parameters and study groups.

Parameter	Value	Total	M group	Control Group	*p*
			N (%)	N (%)	
Oral respiration	Yes	16 (100%)	16 (100.0%)	0 (0.0%)	<0.0005 ^1^
	No	124 (100%)	54 (43.5%)	70 (56.5%)	
Infantile deglutition	Yes	6 (100%)	6 (100.0%)	0 (0.0%)	0.014 ^2^
	No	134 (100%)	64 (47.8%)	70 (52.2%)	
Level of somatic Development	Hypo	25 (100%)	17 (68.0%)	8 (32.0%)	0.136 ^3^
	Normal	107 (100%)	49 (45.8%)	58 (54.2%)	
	Hyper	8 (100%)	4 (50.0%)	4 (50.0%)	
Defective phonation	Yes	23 (100%)	22 (95.7%)	1 (4.3%)	<0.0005 ^1^
	No	117 (100%)	48 (41.0%)	69 (59.0%)	
Slow mastication	Yes	13 (100%)	8 (61.5%)	5 (38.5%)	0.382 ^1^
	No	127 (100%)	62 (48.8%)	65 (51.2%)	
Bruxism	Yes	17 (100%)	11 (64.7%)	6 (35.3%)	0.196 ^1^
	No	123 (100%)	59 (48.0%)	64 (52.0%)	
Salivary aspect	Hypo	21 (100%)	18 (85.7%)	3 (14.3%)	0.002 ^3^
	Normal	110 (100%)	48 (43.6%)	62 (56.4%)	
	Hyper	9 (100%)	4 (44.4%)	5 (55.6%)	
Smoking status	Yes	15 (100%)	5 (33.3%)	10 (66.7%)	0.172 ^1^
	No	125 (100%)	65 (52.0%)	60 (48.0%)	
Consumption of acid drinks	Yes	52 (100%)	25 (48.1%)	27 (51.9%)	0.726 ^1^
	No	88 (100%)	45 (51.1%)	43 (48.9%)	
Thumb sucking	Yes	16 (100%)	16 (100.0%)	0 (0.0%)	<0.0005 ^1^
	No	124 (100%)	54 (43.5%)	70 (56.5%)	
Onychophagia	Yes	29 (100%)	20 (69.0%)	9 (31.0%)	0.022 ^1^
	No	111 (100%)	50 (45.0%)	61 (55.0%)	
Placing objects between the maxillaries	Yes	29 (100%)	29 (100.0%)	0 (0.0%)	<0.0005 ^1^
	No	111 (100%)	41 (36.9%)	70 (63.1%)	
Consumption of seeds	Yes	28 (100%)	13 (46.4%)	15 (53.6%)	0.673 ^1^
	No	112 (100%)	57 (50.9%)	55 (49.1%)	
Frequency of teeth brushing	Never	31 (100%)	15 (48.4%)	16 (51.6%)	0.003 ^3^
	1 time	73 (100%)	46 (63.0%)	27 (37.0%)	
	2 times	30 (100%)	7 (23.3%)	23 (76.7%)	
	3 times	6 (100%)	2 (33.3%)	4 (66.7%)	

^1^ Chi-Square test. ^2^ Fisher Exact test. ^3^ Chi-Square test of homogeneity.

**Table 5:** Distribution of children included in the M group by oral parameters and malocclusion class.

Parameter	Value	Total	Class I	Class II	Class III	*p* ^1^
			N (%)	N (%)	N (%)	
Oral respiration	Yes	16 (100%)	0 (0.0%)	16 (100.0%)	0 (0.0%)	<0.0005
	No	54 (100%)	34 (63.0%)	14 (25.9%)	6 (11.1%)	
Infantile Deglutition	Yes	6 (100%)	6 (100.0%)	0 (0.0%)	0 (0.0%)	0.031
	No	64 (100%)	28 (43.8%)	30 (46.9%)	6 (9.4%)	
Level of somatic development	Hypo	17 (100%)	2 (11.8%)	15 (88.2%)	0 (0.0%)	<0.0005
	Normal	49 (100%)	31 (63.3%)	15 (30.6%)	3 (6.1%)	
	Hyper	4 (100%)	1 (25.0%)	0 (0.0%)	3 (75.0%)	
Defective Phonation	Yes	22 (100%)	9 (40.9%)	12 (54.5%)	1 (4.5%)	0.365
	No	48 (100%)	25 (52.1%)	18 (37.5%)	5 (10.4%)	
Slow Mastication	Yes	8 (100%)	4 (50.0%)	4 (50.0%)	0 (0.0%)	0.642
	No	62 (100%)	30 (48.4%)	26 (41.9%)	6 (9.7%)	
Bruxism	Yes	11 (100%)	10 (90.9%)	1 (9.1%)	0 (0.0%)	0.009
	No	59 (100%)	24 (40.7%)	29 (49.2%)	6 (10.2%)	
Salivary aspect	Hypo	18 (100%)	4 (22.2%)	14 (77.8%)	0 (0.0%)	0.006
	Normal	48 (100%)	27 (56.3%)	16 (33.3%)	5 (10.4%)	
	Hyper	4 (100%)	3 (75.0%)	0 (0.0%)	1 (25.0%)	
Smoking status	Yes	5 (100%)	4 (80.0%)	1 (20.0%)	0 (0.0%)	0.331
	No	65 (100%)	30 (46.2%)	29 (44.6%)	6 (9.2%)	
Consumption of acid drinks	Yes	25 (100%)	20 (80.0%)	3 (12.0%)	2 (8.0%)	<0.0005
	No	45 (100%)	14 (31.1%)	27 (60.0%)	4 (8.9%)	
Thumb sucking	Yes	16 (100%)	0 (0.0%)	16 (100.0%)	0 (0.0%)	<0.0005
	No	54 (100%)	34 (63.0%)	14 (25.9%)	6 (11.1%)	
Onychophagia	Yes	20 (100%)	11 (55.0%)	7 (35.0%)	2 (10.0%)	0.702
	No	50 (100%)	23 (46.0%)	23 (46.0%)	4 (8.0%)	
Placing objects between the maxillaries	Yes	29 (100%)	10 (34.5%)	17 (58.6%)	2 (6.9%)	0.080
	No	41 (100%)	24 (58.5%)	13 (31.7%)	4 (9.8%)	
Consumption of seeds	Yes	13 (100%)	5 (38.5%)	8 (61.5%)	0 (0.0%)	0.223
	No	57 (100%)	29 (50.9%)	22 (38.6%)	6 (10.5%)	
Frequency of teeth washing	Never	15 (100%)	8 (53.3%)	7 (46.7%)	0 (0.0%)	0.189
	1 time	46 (100%)	20 (43.5%)	22 (47.8%)	4 (8.7%)	
	2 times	7 (100%)	4 (57.1%)	1 (14.3%)	2 (28.6%)	
	3 times	2 (100%)	2 (100.0%)	0 (0.0%)	0 (0.0%)	

^1^ Chi-Square test of homogeneity.

## Data Availability

The data presented in this study are available on request from the corresponding author. The data are not publicly available due to privacy.
